# Understanding chronic feelings of emptiness in borderline personality disorder: a qualitative study

**DOI:** 10.1186/s40479-021-00164-8

**Published:** 2021-08-09

**Authors:** Caitlin E. Miller, Michelle L. Townsend, Brin F. S. Grenyer

**Affiliations:** 1 School of Psychology, University of Wollongong, Building 22, NSW 2522 Wollongong, Australia; 2 Illawarra Health and Medical Research Institute, University of Wollongong, NSW Wollongong, Australia

**Keywords:** Emptiness, Borderline personality disorder, BPD, Self-harm, Suicide, Impulsivity, Depression, Hopelessness, Loneliness

## Abstract

**Background:**

Chronic feelings of emptiness are significant in the lives of people with Borderline Personality Disorder (BPD). Feelings of emptiness have been linked to impulsivity, self-harm, suicidal behaviour and impaired psychosocial function. This study aimed to understand the experience of chronic emptiness, the cognitions, emotions and behaviours linked to emptiness, and clarify the differences between chronic emptiness and hopelessness, loneliness and depression.

**Methods:**

This study interviewed people (*n* = 15) with BPD and used a template analysis qualitative approach to understand their experiences of chronic feelings of emptiness.

**Results:**

Chronic feelings of emptiness were experienced as a feeling of disconnection from both self and others, and a sense of numbness and nothingness which was frequent and reduced functional capacity. Feelings of purposelessness and unfulfillment were closely associated with emptiness, and most participants experienced emptiness as distressing. Responses to feelings of emptiness varied, with participants largely engaging in either impulsive strategies to tolerate feelings of emptiness or distracting by using adaptive behaviours. Most participants distinguished chronic feelings of emptiness from loneliness, hopelessness, dissociation, and depression.

**Conclusions:**

Feelings of chronic emptiness are an important and challenging symptom of BPD which require clinical intervention. Strengthening identity, sense of purpose and vocational and relationship functioning may reduce the intensity of emptiness.

**Supplementary Information:**

The online version contains supplementary material available at 10.1186/s40479-021-00164-8.

## Background

Borderline personality disorder (BPD) is characterised by pervasive distress and dysfunction in self and interpersonal spheres [1, 2]. Within both categorical and dimensional models of classification, chronic feelings of emptiness is included as a symptom of BPD. In the alternate model of diagnosis in the Diagnostic and Statistical Manual, Fifth edition (DSM-5), chronic emptiness is understood as a component of unstable self-identity alongside self-criticism and dissociative experiences [1]. Historically there has been sparse empirical research into chronic feelings of emptiness [3] – perhaps due to the perceived difficulty in defining and measuring what is assumed to be an absence of experience, and the focus on more acute symptomology. However, there have been recent efforts to increase the understanding of chronic emptiness, with researchers defining it as comprising a feeling of detachment and disconnection from both self and other people [4].

Chronic feelings of emptiness are significant to the conceptualisation, course and outcomes of BPD. Theoretical models have linked the experience of chronic emptiness to deficits in self- and object-representations, resulting in a diffuse or unstable identity and disconnection from other people [5–7]. Biosocial models propose that individuals with BPD experience emotional sensitivity and reactivity, in addition to deficits in regulating intense emotions. These models suggest the inability to understand and tolerate internal experiences at times can lead to individuals inhibiting any emotional experience, and an associated feeling of emptiness inside [8, 9]. It is hypothesised that people with BPD may engage in maladaptive behaviours to ‘fill up’ the emptiness they experience as distressing [10] and qualitative accounts of women with BPD highlight the relationship between chronic feelings of emptiness and identity disturbance [11]. Feelings of chronic emptiness have been associated with aversive experiences and poor outcomes over time. Chronic emptiness has been linked with impulsivity [10], self-harm [12, 13], and suicidal behaviour [14] and is predictive of impaired function both socially and vocationally [10, 15]. Chronic emptiness has been considered one of twelve temperamental symptoms of BPD (alongside other predominant symptoms e.g. chronic depression, feelings of helplessness and hopelessness, abandonment fears), which compared to more acute symptoms (e.g. affective instability, self-injury, unstable relationships) are slow to resolve over time with low remission and high recurrence rates over 16 years [16].

Although research into chronic emptiness is beginning to develop an understanding of the aetiology, features, and impact of chronic emptiness in BPD, there are still important gaps in the field. Firstly, there remains no consensus on the phenomenological experience for people with BPD. While chronic emptiness emerged as a theme of life stories for women with BPD [11], there have been no studies to the authors’ knowledge specifically trying to understand the nature of chronic emptiness in BPD. Secondly, the available data remains unclear on the boundaries between chronic emptiness and related constructs. Studies have suggested there may be distinguishable features of chronic emptiness, hopelessness, loneliness and depression [4, 13, 17], however no studies have explicitly attempted to differentiate these experiences by asking people with lived experiences of BPD. To capture the phenomenological experience of chronic emptiness, there is a need for qualitative accounts of people with lived experiences of chronic emptiness in BPD.

### The current study

To address the gap in the research, this research aims to capture the phenomenological experiences of chronic emptiness for individuals with BPD. Specifically, the research aims to identify common experiences of chronic feelings of emptiness for people with BPD, understand cognitions, emotions and behaviours linked to emptiness, and clarify the differences between chronic emptiness and related experiences.

## Method

### Participants

Selective sampling methods identified participants suitable for this study from those enrolled in a larger ongoing longitudinal study, which is described elsewhere [10]. In brief, participants have been followed with phone interviews every nine months since participating in interventions following presentation to health services. Participants who had endorsed feeling chronically empty ‘a good bit of the time’ or more in the past fortnight when asked about symptoms of BPD in at least one previous interview were invited to participate. Forty-one participants were contacted to participate in the study. Twenty were deemed uncontactable following unsuccessful contact attempts, five participants declined to participate, one participant consented but was uncontactable for the interview. Fifteen participants completed the study, and all interviews were analysed. All participants gave explicit consent following Institutional Review Board approval.

### Procedure and measures

Participant’s interview included administration of the BPD component of the Structured Clinical Interview for DSM-5 (SCID-II) and a semi-structured interview regarding common experiences, cognitions and behaviours and related experiences of chronic emptiness (see Additional File 1). Interviews were audio recorded, transcribed verbatim and entered into NVivo 12 Plus for analysis.

#### Severity of chronic emptiness

Participants were asked to rate ‘how often have you felt chronically empty in the last two weeks?’ on a scale of 1 (none of the time) to 6 (all of the time).

#### Structured clinical interview for DSM-5 (SCID-II), BPD section

The SCID-II [18] was utilised to determine presence of BPD symptoms. The SCID-II is a semi-structured interview for personality disorders with questions based upon DSM-5 symptoms with satisfactory internal consistency [19]. To reduce participant burden, only the questions of the BPD section of the SCID-II were asked.

### Data analysis

A template analysis approach was utilised for qualitative transcripts, underpinned by a critical realism ontological approach which explores lived experiences to interpret and make meaning of underlying mechanisms or phenomena [20]. Template analysis represents a codebook approach to thematic analysis which combines the qualitative philosophy of reflexivity and a more structured technical approach of coding [21]. This approach was chosen given the flexibility of the method, allowing the use of both inductive and deductive knowledge to develop in-depth explanations and meaning [21–23]. This enabled the use of some apriori themes to guide researcher focus in an area without significant existing research, while also allowing the phenomenological experience of individuals to shape and develop the coding [24]. Accordingly, an initial codebook was developed from the literature that contained possible codes. Five transcripts were studied by one researcher in an iterative process that resulted in revised codes and new inductive codes emerging from the interviews. Following this, a second researcher coded five transcripts independently using the code manual, then codes were discussed and refined between researchers until agreement was achieved. The remaining transcripts were analysed using this codebook, with further revision of the codebook where necessary following discussion between researchers. After 15 interviews were analysed, no new information or themes were emerging from the data, and it was decided further recruitment and interviews were not necessary. Research seeking to understand the experience of individuals is shaped by researchers’ own experiences [25], and a reflexive process was used throughout data analysis including the independent analysis of some transcripts and discussion between researchers.

## Results

### Participant demographics and clinical characteristics

In the final sample of 15 participants, the mean age was 37.42 (*SD* = 12.35, range = 24–63 years), and 80 % of the sample were female (*n* = 12). All participants had sought treatment from a psychologist, psychiatrist or both in the past two years. The average rating of chronic emptiness over the past fortnight was 3.4 (*SD* = 1.68, range = 1–6). Demographic and clinical information is provided in Table 1.

**Table 1:** Demographic and clinical variables of participants

Variable	Number of participants (%)
Employment	
Full-time	6 (40)
Part-time	2 (13.3)
Disability pension	3 (20)
Dependent on others	1 (6.7)
Caring for others	1 (6.7)
Unemployed	1 (6.7)
Temporary benefit	1 (6.7)
Relationship status	
Single	7 (46.7)
Married	4 (26.7)
In a relationship	3 (20)
Separated	1 (6.7)
Academic qualifications	
Post-high school degree	6 (40)
Post-high school diploma	6 (40)
High school graduate	1 (6.7)
Did not graduate high school	1 (6.7)
Did not respond	1 (6.7)
Chronic emptiness in past fortnight	
None of the time	3 (20)
A little of the time	1 (6.7)
Some of the time	4 (26.7)
A good bit of the time	3 (20)
Most of the time	2 (13.3)
All of the time	2 (13.3)

Following administration of SCID-II, 93.3 % of the sample (*n* = 14) currently met criteria for five or more symptoms of BPD. Participants most commonly endorsed abandonment fears (93.3 %), affective dysregulation (93.3 %), identity disturbance (80 %) and chronic emptiness (80 %). One participant endorsed three symptoms of BPD, however had previously met threshold for a diagnosis and three participants noted they did not currently experience feelings of chronic emptiness. These participants were able to reflect on their past experiences.

The four participants who endorsed the highest severity (most or all of the time) of chronic emptiness were all single, not currently employed and had an average age of 50 years. The four participants who endorsed the least severe emptiness in the past fortnight were all in a relationship or married, and were in part- or full-time employment or caring for others. The average age of these participants was 31 years.

### Identifying common experiences of chronic feelings of emptiness for people with BPD

#### Difficulties describing the phenomenon of chronic emptiness

Typically, participants found it difficult to articulate the experience of chronic emptiness to the interviewer, however, many participants were able to think of a metaphor for how chronic emptiness feels – see Table 2. These metaphors were able to provide a rich understanding of the nature of chronic emptiness.

**Table 2:** Metaphors for the experience of feeling chronically empty

219,064	*So, I guess, emptiness is like an overcast day where it’s − but it’s a chill on the wind, but it’s not aggressively windy. Um, it’s just, you know, you can’t get warm, um, and you can’t, sort of, find a nice warm spot everywhere you go, whether it’s, like, out in the open, or under, you know, not being under a tree or anything. It’s just like, just like constantly cold. But it’s not raining or miserable weather. It’s just sort of overcast and a bit chilly.*
219,065	*It’s just your kind of very robotic and very, like − there is no meaning or purpose, I guess, would be my definition of it.*
219,074	*Oh dear, it’s a black hole… Um, but I guess it’s that drowning, you know, drowning.*
219,088	*It’s like wind inside a tin can… like you’re the tin can.*
229,070	*Um, um… Mmm. Like a stone that hasn’t been shaped into a rock.*
259,029	*Um, nothingness. Nothingness as in no sense of being. Um - - - like, I suppose, like, with the cosmos, like a black hole or something. I suppose, like, um, there’s no sense of time. What do you call it? You know how the astronauts, when you see them walking around in space and there’s no gravity? That sense. The sense of not being able – the sense of not, of no, of no – the sense of weightlessness probably or – I don’t know, I don’t know.*
259,090	*Um, I suppose like just a depressing, empty swimming pool. Like a concrete swimming pool with, like, a little bit of water and lots of mould… Yeah. Like not one that people skate in.*

*Note –* Metaphors were prompted by the question “Can you think of a metaphor that describes how you’ve experienced feeling empty inside?”

#### Sense of nothingness, numbness and disconnection from self and others

Participants largely defined feelings of chronic emptiness as a sense of nothingness or a feeling of numbness. #219,132 *“Numb. It reminds me of a dead leg, kind of. Like, the sensation of the fuzziness is there.”* #249,020 remarked *“It’s like being in a dark room. And you’re just sitting in the middle of a completely dark room. And there’s nothing.”* Participants likened feelings of chronic emptiness to a feeling of disconnection from themselves. This included feeling they have no identity and feeling like their identity is unstable. For participants who described emptiness as an absence of self, they reported it was a sense of not feeling like they are a person. #259,029 remarked *“It’s like a sense of not-being, like because I’m no being, a sense of no body… to me, identity means you’re a person, to me emptiness is not a person. When I feel like there’s some emptiness I have – I’m not a person. I don’t feel like I’m a person.”* Participant #259,004 described emptiness in a similar way, *“Like there’s nothing, there’s nothing there, that there’s no, like, there’s no emotion, there’s no, there’s no me… Yeah. Emptiness I just feel like there’s nothing left of me”* Similarly, a participant reported their feelings of emptiness were linked to a diffuse sense of identity, and related this to feeling like a chameleon who changes colours according to the situation.

Some participants also described chronic feelings of emptiness as a sense of disconnection from others, though this was described less often than a disconnection with self. Participant #259,029 remarked *“*[chronic emptiness is] *disconnection from people. It’s like, I’m looking, I’m an observer as opposed to participant”.* One participant noted that chronic emptiness occurs when they are *“not connecting with people, and being, like, kind of − I have got a tendency to just, like, stay in my room and, just, like, avoid the world. So, if I’m kind of doing those − like, if I’m kind of doing negative behaviours, I find that the emptiness comes”* (#219,065). Other participants noted that chronic emptiness often arose when they were experiencing interpersonal distress – such as comparing themselves to others or feeling like no one cared about them, or interpersonal dysfunction – including conflict in relationships.

#### Feelings of unfulfillment and purposelessness associated with emptiness

Chronic emptiness was often related to cognitions and emotions of purposelessness and unfulfillment. One participant reflected emptiness resulted in a *“purposeless kind of living. It’s just your kind of very robotic and very, like – there is no meaning or purpose”* (#219,065). For some participants, this feeling of purposelessness was characterised by low agency, and a lack of self-direction, values and goals. #259,049 stated *“I didn’t really know what to do; going for jobs, application processes but, you know didn’t go through… I was sort of doubting or questioning my life at that point.”* While #259,090 remarked *“I just felt that I was going through the motions of life I suppose and didn’t actually, um, enjoy life at all for a little time.”* This sense of purposelessness often culminated in a feeling of unfulfillment – participant reports included *“not living, a life of fulfilment… there’s something missing, I’m not living the life that I want to live… why… why, what am I doing right now, like why… What am I doing with my life that is fulfilling me right now?”* (#229,070), *“I don’t feel like there’s anything worth getting out of bed for”* (#219,088) and *“questioning the whole point of life and existence”* (#229,110).

#### Feelings of emptiness are enduring, frequent and difficult to alleviate

 Participants discussed the nature of emptiness as being chronic and difficult to alleviate. One participant noted they did not know what it was like to not feel empty anymore, and others reported a feeling of being stuck with nothing changing. Participants also described emptiness as a frequent experience but noted that it was not always constant and will *“come and go”* (#229,072). Participants found it difficult to identify what helps to alleviate emptiness in the long-term, but had developed short-term preventative and coping strategies. One participant likened the experience of chronic emptiness to the descriptions of Dementor’s in Harry Potter (hooded wraithlike creatures that consume a human’s soul until there is only despair left; [26]). She reported that *“the Dementor’s over you and you, like I said before, you as much as you want to try and beat it there’s – it’s just, you’re lifeless. So you – you really can’t do anything to, sort of, overcome that”* (#219,112).

#### Chronic emptiness occurs when not distracted

Participants reported chronic emptiness most often occurred when they were not busy or distracted. One participant #259,004 said *“it can just be, like, any random day, um, it doesn’t have to be a trigger or anything… I can just wake up feeling like that.”* Similarly, other participants reflected chronic emptiness arose when they were not making an effort to connect with the people close to them, when they were sitting in traffic and most commonly following the completion of daily activities and prior to sleep. Interestingly, several participants commented on employment functioning as a distraction from the feeling of emptiness. One participant reported *“Even though I might, um, be doing quite well, just coming back to myself at the end of the night and then just being in my own, um – in my own thoughts… throughout the day I’m quite okay because I do have that, um – I have that sense of being able to distract myself through other – other, um, areas, either work, um, gym, my friends or my family… It’s when I get home at the end of the night”* (#219,112).

#### Emptiness reduces functional capacity

A further theme that emerged was reduced capacity or inability to function when experiencing emptiness. Participants often reported this when differentiating chronic emptiness from similar experiences. One participant noted that *“emptiness – it’s just, like, it’s just, yeah, it stops you dead in your tracks, you know… just stops you and you don’t want to go out the door*.*”* (#219,074). Other participants reported *“I didn’t do much in those two years. Like, I did not have much of a life, it was quite a blur”* (#219,132) and *“with the emptiness I find it really hard to function”* (#259,004).

### Understanding cognitions, emotions and behaviours linked to emptiness

#### Chronic emptiness as distressing versus the use of chronic emptiness as distress tolerance

 Typically, participants spoke about wishing they could not feel empty and trying to create feeling when they do experience emptiness. These participants indicated that the experience of chronic emptiness was distressing for them, and they actively try to avoid the experience. Some participants, however, reported that they use chronic emptiness as a distress tolerance strategy. They spoke about wanting to feel empty inside rather than feel an intense emotion and out of control. This seemed to represent an active choice to inhibit intense emotion, as #219,065 reported *“if it’s like way too intense, I will just shut it down because I can’t deal with it in that moment”*.

#### Preventative, coping and alleviating strategies for emptiness

 Participants discussed a wide range of ways in which they try to prevent chronic emptiness from occurring, and methods for coping and alleviating chronic emptiness. Typically, similar strategies were employed to prevent, cope with, and alleviate chronic emptiness.

Maladaptive strategies included impulsive behaviours and rumination on thoughts of purposelessness and unfulfillment. Participants spoke about generating alternate affects to emptiness through impulsive behaviours such as self-harm, #219,065 *“If I’m feeling empty and I’m feeling like, oh well, I just don’t want to sit in this space, I just want something to counteract that, so I’m just going to do something impulsive.”* These participants also endorsed avoidance of cognitions associated with emptiness, however reported this avoidance can also result in impulsive or unhelpful behaviours. For individuals who engaged in maladaptive behaviour, they more often reported they had not thought about how impulsive behaviours may be an attempt to alleviate feelings of chronic emptiness prior to the interview, but when prompted felt that they were linked.

For participants who had more adaptive methods of preventing, coping and alleviating chronic emptiness, the most common method of tolerating and alleviating chronic emptiness was through utilising behavioural activation skills. These participants spoke of an active choice to engage in a different behaviour to alleviate emptiness. Several participants reported that when these behaviours are unsuccessful, they resort to sleep in the aim of ‘resetting’ themselves. Cognitively, some participants reported they choose to ignore or delay focusing on cognitions and emotions associated with emptiness until it abates, participant #219,112 remarked *“[I] just involve myself in everything just to keep my mind distracted”*. However, these participants often noted that avoiding cognitions was only a temporary solution. The distinguishing feature of adaptive coping was awareness of the emotion, which appeared to provide an opportunity to choose a coping strategy rather than engage in an immediate reaction.

### Clarifying the differences between chronic emptiness and associated experiences

When asked to reflect on if chronic feelings of emptiness were distinguishable from experiences of depression and loneliness, most participants identified emptiness as a distinctive experience. Participants indicated the distinguishing feature was that depression included distressing feelings and cognitions, while emptiness felt like an absence of everything. Participant #219,075 stated *“depression is more thinking and emptiness is lack of thinking… your mind is not processing it; it’s just empty”* while participant #219,132 reported *“depression, um, to me, is an emotion. Um, to me, it is sadness… whereas emptiness is nothing… emptiness is so neutral”*. Similarly, participant #229,110 remarked *“No, it is different, depression is just you feel like you’re in a… whirlpool or a crevasse and you’re just falling and, there’s no getting back. Emptiness is just nothing.”* Interestingly, some participants reflected that it was more difficult to function when feeling empty compared to when feeling depressed, possibly indicating that the absence of any thought or feeling results in feelings of anhedonia and amotivation beyond those feelings present in depression. Participants also distinguished between chronic emptiness and feelings of loneliness. Several participants noted that loneliness and chronic emptiness can coincide, and loneliness can often precede feelings of emptiness inside. In a similar way to depressive experiences, a distinguishing feature of loneliness included that *“loneliness, like it is an actual feeling, like − and emptiness is just like lack of feeling”* (#219,075), and participants reported they could still function when feeling lonely. Participants attributed feelings of loneliness to a lack of social connections, while chronic emptiness was characterised by a disconnection from both self – goals, ideas, and values – in addition to disconnection from other people. The disconnection from others was not necessarily due to a lack of relationships, but rather *“not feeling like you’re connected”* (#219,088). Further, participants indicated that loneliness could be alleviated by distraction or social connection, whereas emptiness was more debilitating – one participant reflected *“emptiness is like, what I feel about myself… loneliness can be cheered by meeting up with people*”. Some participants spontaneously linked the experience of chronic emptiness to hopelessness. One participant reported chronic emptiness and hopelessness were the same experience, however, other participants noted that chronic emptiness is an absence of feeling while hopelessness is *“the dread feeling”* (#219,074). The common theme among the comparisons between chronic emptiness and associated experiences was that feelings of emptiness were characterised by an absence of thoughts and feelings, rather than the presence of distressing experiences. Interestingly, four participants also spoke of the differences between emptiness and dissociation. They reported that they disconnect or ‘zone out’ when experiencing feelings of emptiness. This was described as a dream-like state, not being in tune with surroundings, and not paying attention. These participants reported it was similar but distinguishable from dissociation, as #259,004 said *“I still know that, like, time’s passing, and afterwards I can tell you what I did during that time, but I’m still just sitting there, and I’m just not paying attention, like I’m just zoned out, rather than dissociated.”*

## Discussion

This study aimed to understand how individuals with BPD experience the feeling of chronic emptiness. This included clarifying the nature of emptiness, identifying common experiences, cognitions, emotions and behaviours linked to chronic emptiness and understanding the differences with associated constructs. Chronic emptiness was largely reported as a unified construct, with most participants describing a sense of nothingness and numbness that represents a feeling of disconnection with self and others and resulted in feelings of unfulfillment and purposelessness. These findings echo descriptions of emptiness as characterised by low positive affect which creates significant distress rather than intense negative affect [13, 27]. Feelings of chronic emptiness were frequent but not constant, difficult to alleviate and reduced an individual’s capacity to function effectively. Chronic emptiness was significant in the lives of participants and impacted their cognitions and emotions regarding themselves, other people and the world.

Feelings of chronic emptiness in this sample seemed to stem from a disconnection from self and others. In relation to a disconnected sense of self, participants noted that chronic emptiness arose from feeling like they had no identity, that their identity was unstable and difficulties with self-direction, values and goals. This supports previous theoretical work that considers emptiness in part as a reflection of disturbed self-representations [6, 28, 29]. Furthermore, participants reported that a disconnection from self and an associated feeling of emptiness often led to feelings and cognitions of purposelessness in life.

 Participants also discussed a disconnection from others as a source of chronic emptiness, which often occurred in the context of interpersonal distress and dysfunction. Theoretically, this may relate to impaired other-representations, experiences of invalidation and difficulties with internalising positive social experiences [6, 9]. A feeling of disconnection from others may be reflective of the epistemic distrust, hypermentalising and other social cognition deficits that are present for people with BPD, and the relationship to disturbances in identity [30–33]. If this is so, chronic emptiness may both arise from deficits in social cognition and identity, and perpetuate these difficulties where people experience a sense of nothingness in themselves, their relationships to others and in the world – thus possibly limiting their ability to connect with others and disrupt the feeling of emptiness.

For most participants, chronic emptiness was experienced as distressing and they attempted to prevent, tolerate or alleviate chronic feelings of emptiness. Participants who had not previously considered the link between emptiness and impulsive behaviours prior to participation in this research often attempted to relieve emptiness by engaging in maladaptive and impulsive coping strategies. This supports previous literature that hypothesised emptiness feels intolerable and people engage in impulsive behaviours to generate alternate affects to emptiness [10, 13, 15]. It may also indicate maladaptive responses could arise from difficulties with identifying emotions and their behavioural sequalae. On the other hand, participants who had already identified a link between emptiness and urges to engage in impulsive behaviour discussed noticing feelings of chronic emptiness and choosing to engage in adaptive behaviours to alleviate or tolerate the feeling. Perhaps, engaging in a form of activity aids in quelling or distracting from the emotional and cognitive load of emptiness and those that were more aware of the experience were able to make an active choice on how to respond. This is a novel finding with clinical relevance. Firstly, clinicians may benefit from looking beyond impulsive or self-destructive behaviours and exploring what experience spurs these behaviours. This may increase awareness, reflective capacity and mindfulness of emotion. Secondly, when clients are experiencing difficulty with chronic feelings of emptiness, clinicians may work collaboratively with clients to determine adaptive coping strategies. Specifically, it seems that engagement in vocation and relationships may serve as both a protective buffer against disconnection from self and others and subsequent emptiness. While strategies including behavioural activation may be helpful in replacing maladaptive strategies for coping with chronic emptiness, most participants noted that they are a short-term strategy. Participants were unsure what helps emptiness to resolve in the long-term, which may reflect both the chronic nature of emptiness and the lack of treatment targeted towards the experience.

 While most participants reported they found feelings of emptiness distressing, some participants noted chronic emptiness could be brought on intentionally to tolerate distress, in an attempt to regulate intense emotion and prevent behavioural dyscontrol. This may be important in clinical practice for clinicians to discern how individuals relate to feelings of chronic emptiness and the distress associated with emptiness.

Most participants differentiated between feelings of chronic emptiness and associated constructs like loneliness, hopelessness and dissociation. The distinguishing factor was that chronic emptiness is an absence of emotion compared to other experiences which have a visceral feeling. However, participants noted that emptiness and loneliness often coincide which is unsurprising given that the experience of loneliness for people with BPD is prevalent and persistent [34]. It is possible that the disconnection from others experienced as chronic emptiness may be exacerbated by feelings of loneliness.

The majority of participants indicated their experience of chronic emptiness could be differentiated from the experiences of depression. While a meta-analysis reported support for a BPD-specific depression characterised by anger, hostility and self-criticism, there were no studies that compared feelings of emptiness in BPD and depressive disorders [17]. This study has shown that from a qualitative perspective, chronic emptiness and depression may be related but are separate experiences for this sample of people with BPD.

This study has several limitations. Participants had all received psychological intervention for BPD within the past two years and not all participants were experiencing severe levels of chronic emptiness at the time of the interview. While this enabled some participants to reflect meaningfully and articulately describe their past experiences, it is possible that chronic emptiness is experienced differently for those who do not receive treatment or who are more acutely symptomatic. Similarly, the results of this study were dependent upon participants being able to articulate an internal experience most commonly defined as an absence of feeling. This was a challenge for some participants and may have resulted in a reliance on participants who were able to effectively articulate their experience of emptiness – most often those who were experiencing emptiness less severely or speaking about past experiences of emptiness. Interview questions directly asked about the link between identity and chronic emptiness, however did not further extrapolate between different forms of identity such as social- and self-identity. As with all qualitative research, interpretation of the participant interviews is influenced by researcher assumptions and expectations which may lead to researcher bias [35].

Future studies could investigate the role of social cognition deficits in identity and chronic emptiness and explore emotion awareness and the impact on behaviour in BPD. Also, more knowledge is required to better understand the differences between people with BPD who actively try to inhibit their emotions by creating a feeling of emptiness, versus those for whom chronic emptiness is unavoidable and distressing. Similarly, future research could further investigate the similarities, differences and relationship between dissociation and chronic feelings of emptiness. While participants in this study provided a range of short-term measures to prevent, tolerate and alleviate chronic feelings of emptiness, the field may benefit from studies trying to understand how to reduce the severity and impact of chronic emptiness over the longer-term for people with BPD. As chronic emptiness is one of the last symptoms of BPD to resolve [16], research into interventions for chronic emptiness is warranted.

## Conclusions

This novel study found that for people with BPD, chronic emptiness is experienced as a sense of nothingness and numbness that reflects a feeling of disconnection from both self and others. It is associated with feelings of unfulfillment and purposelessness. Chronic emptiness is a frequent experience that significantly limits the functional capacity of people with BPD and is distinguishable from loneliness, hopelessness, dissociation, and depression. It is possible that reduction in identity disturbance and improved vocational and relationship functioning may reduce the intensity of chronic emptiness. It is difficult to alleviate, however there are a range of strategies people with BPD engage in to prevent, tolerate and alleviate chronic emptiness which may be harnessed for future interventions.

## Supplementary Information

**Additional file 1.**

## Data Availability

The datasets analysed during the current study are not publicly available but are available from the corresponding author on reasonable request.
