# Supplementary material for: Understanding chronic feelings of emptiness in borderline personality disorder: a qualitative study
Source: Borderline Personal Disord Emot Dysregul. 2021 Aug 9;8:24. doi: 10.1186/s40479-021-00164-8 (PMC8351135; doi:10.1186/s40479-021-00164-8)
Supplement: Supplementary file 1 — Additional file 1. [file 40479_2021_164_MOESM1_ESM.docx]

Additional file 1

1. In the past two weeks, how often have you felt chronically empty (1 = none of the time through 6 = all of the time)
2. Describe a typical experience of a time when you felt empty. Try to get as detailed as possible so I understand exactly how it felt for you and what you were thinking and feeling
3. Describe any times you’ve deliberately tried to feel empty
4. Are feelings of emptiness the same as feelings of loneliness?
5. For you, are feelings of emptiness different to feelings of depression?
6. Have feelings of emptiness ever manifested in impulsive behaviours (including self-harm)?
7. Do you think feelings of emptiness may be linked to your sense of identity?
8. If you had to describe emptiness as a colour, what colour would it be?
9. Can you think of a metaphor that describes how you’ve experienced feeling empty inside?
10. What do you do when you feel empty inside?
11. Tell me about any times and ways you’ve tried to avoid the experience of feeling empty
